# Short-term storage of semen samples in acidic extender increases the proportion of females in pigs

**DOI:** 10.1186/s12917-021-03078-3

**Published:** 2021-11-26

**Authors:** Yoo-Jin Park, Dong-Ha Shin, Won-Ki Pang, Do-Yeal Ryu, Md Saidur Rahman, Elikanah Olusayo Adegoke, Myung-Geol Pang

**Affiliations:** Department of Animal Science & Technology and BET Research Institute, Chung-Ang University, 4726 Seodong-daero, Deadeok-myon, Anseong, Gyeonggi-do 17546 Republic of Korea

**Keywords:** Artificial insemination, Boar spermatozoa, Acidic semen extender, Sex ratio, Sex preselection

## Abstract

**Background:**

Sex preselection is a desired goal of the animal industry to improve production efficiency, depending on industry demand. In the porcine industry, there is a general preference for pork from female and surgically castrated male pigs. Therefore, the birth of more females than males in a litter leads to economic benefits and improved animal welfare in the pig production industry. Our previous study suggested that the porcine semen extender (BTS) adjusted to pH 6.2 maximises the differences in viability between X-chromosome-bearing (X) spermatozoa and Y-chromosome-bearing (Y) spermatozoa without affecting sperm’s functional parameters. In this study we aimed to evaluate whether the pH 6.2 extender is applicable at the farm level for increasing the number of female piglets without a decline in spermatozoa fertility. Artificial insemination (AI) was carried out with spermatozoa stored at pH 6.2 and pH 7.2 (original BTS) at day 1 and day 2 of storage. Next, the functional parameters of the spermatozoa, litter size, farrowing rate, and female-to-male ratio of offspring were determined.

**Results:**

Although sperm motility decreased significantly after 2 d of storage, the viability of spermatozoa was preserved at pH 6.2 for 3 d. There was no significant difference in the farrowing rate and average litter size between the group inseminated with the spermatozoa stored in (pH 7.2) and that inseminated with spermatozoa stored in acidic BTS. The percentage of female piglets was approximately 1.5-fold higher in sows inseminated on day 1 in the pH 6.2 than in the pH 7.2 group. Furthermore, although there was no significant difference in the female-to-male ratio, the percentage of female piglets born was slightly higher in the pH 6.2 group than in the pH 7.2 group on day 2.

**Conclusions:**

The method optimised in our study is simple, economical, and may enhance the number of female births without any decline in spermatozoa fertility.

## Background

In the livestock production industry, sex preselection is desirable to improve reproductive efficiency for specialised purposes such as milk production, breeding for artificial insemination (AI), and meat production. Pork production relies on meat from female and castrated pigs rather than that from boars, despite the cost-effectiveness of raising entire boars due to their higher feed conversion efficiency and growth rate, because 5–10% of the meat from boars has an unpleasant meat taint, which affects meat acceptability [1]. As surgical castration is a debatable option in terms of animal welfare, alternatives to castration or a new technique for producing a higher number of females is required [2]. Over the past decades, several methods based on nutrition, weather, stress, hormone levels, insemination time, and location of insemination in the female reproductive tract have been used to manipulate the sex ratio of offspring; however, the efficiency of sex preselection using these methods remains debatable [3–6]. Recently, AI was performed using sexed spermatozoa or embryos sorted by flow cytometry, which showed more than 90% accuracy in sex preselection [7, 8]. Although this method can produce the desired offspring with high probability, it has some limitations for field applications such as low sperm concentration for boar AI and severe damage to cells during the staining and sorting procedures [9]. Furthermore, this method requires expensive equipment and skilled experts [10]. Therefore, the development of simple and inexpensive methods for the preselection of porcine offspring at the farm level is required to enhance profits in the pig industry.

Previously, we have reported that Y-chromosome-bearing (Y) spermatozoa are more susceptible to stress, such as endocrine disruptors, pH, incubation time, and temperature, compared to the X-chromosome-bearing (X) spermatozoa in humans and mice [11–14]. We also found that the X:Y ratio of live porcine spermatozoa stored in the acidic (pH 6.2) porcine semen extender (BTS) for 2 d was maximised without affecting the intrinsic fertilising ability of spermatozoa [15]. However, no effort has been made to integrate this process at the farm level. Therefore, in this study we aimed to evaluate whether this modified acidic BTS can be applied to porcine AI techniques at the farm level to increase the number of female piglets without negative fertility outcomes.

## Results

### Effect of pH on sperm functional parameters

**Fig. 1:**
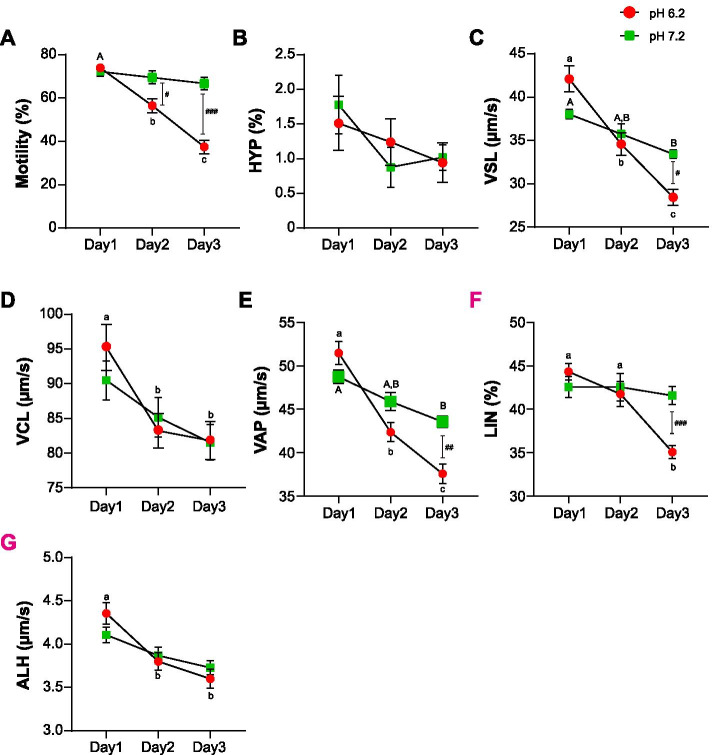
Changes in sperm fertilising parameters after storage at different pH levels. Differences in (**A**) percentage of sperm motility, (**B**) percentage of hyperactivated (HYP) sperm motility, (**C**) straight line velocity (VSL), (**D**) curvilinear velocity, (**E**) average path velocity (VAP), (**F**) linearity (LIN), and amplitude of lateral head displacement (ALH) after storage under different pH conditions. Data represent mean ± SE. ^A-B^*P* < 0.05 versus 1 d at pH 7.2. ^a-b^P < 0.05 versus 1 d at pH 6.2. ^#^P < 0.05, ^##^*P* < 0.01, ^###^*P* < 0.001 versus pH 7.2

To evaluate the changes in sperm functional ability in the pH 7.2 and pH 6.2 semen samples after 3 d, sperm viability, motility, and motion kinematics were evaluated. Sperm motility decreased drastically after day 2 at pH 6.2 (*p < 0.05*), whereas there was no significant change at pH 7.2 on 3 d. Therefore, motility was lower at pH 6.2 than that at pH 7.2 (Fig. 1A, *p < 0.05*). HYP was identified as spermatozoa have curvilinear velocity (VCL) > 80 μm/s, linearity (LIN) < 65%, and an amplitude of lateral head displacement (ALH) > 6.5 μm. There was no significant difference in HYP in either group over 3 d (Fig. 1B). Straight line velocity (VSL) significantly declined from day 2 at pH 6.2, whereas VSL decreased from day 3 at pH 7.2. The VSL was significantly lower at pH 6.2 than that at pH 7.2 after day 3 (Fig. 1C, *p < 0.05*). Although VCL decreased significantly from day 2 at pH 6.2 (*p < 0.05*), there was no difference between pH 6.2 and pH 7.2 during the 3 d (Fig. 1D). Although the average path velocity (VAP) of spermatozoa decreased significantly from day 2 at pH 6.2, a significant decline was observed from day 3 at pH 7.2 (*p < 0.05*). On day 3, the VAP of spermatozoa at pH 6.2 was significantly lower than that of spermatozoa at pH 7.2 (Fig. 1E, *p < 0.05*). While the LIN was significantly declined from day 3 at pH 6.2, there was no difference in pH 7.2 for 3 days (Fig. 1F). Consequently, LIN was significantly lower at pH 6.2 than that at pH 7.2 after day 3 (Fig. 1F). The VCL decreased significantly from day 2 at pH 6.2, whereas no differences were detected at pH 7.2 for 3 days (Fig. 1G).

**Fig. 2:**
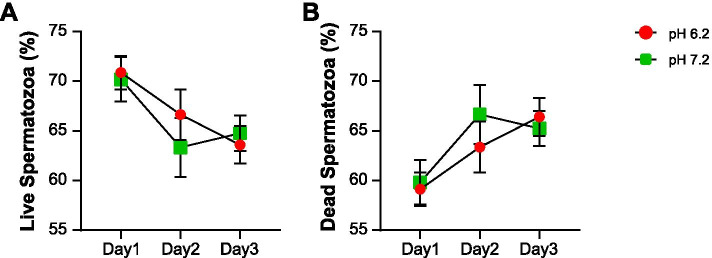
Changes in viability of spermatozoa during storage at different pH levels. Percentage of (**A**) live and (**B**) dead spermatozoa during storage at different pH levels. Data represent mean ± SE

The changes in live/dead spermatozoa were not significant in either group and there was also no difference in these changes between the groups (Fig. 2).

**Fig. 3:**
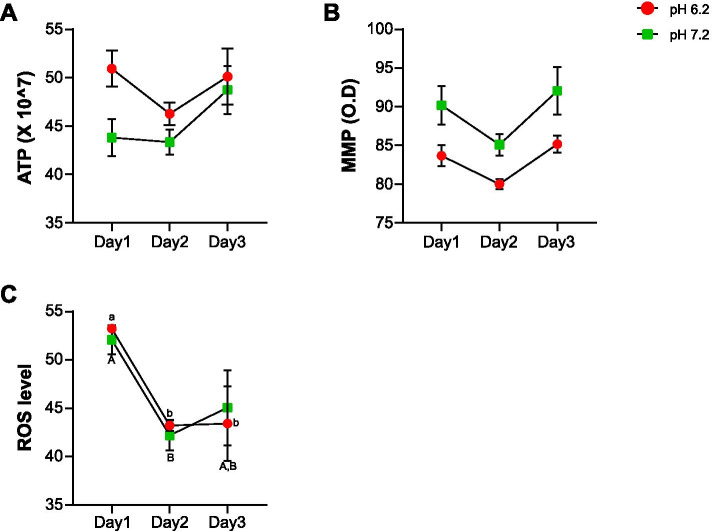
Effect of different pH levels on sperm functional parameters. Changes in (**A**) intracellular ATP, (**B**) mitochondrial membrane potential (MMP), and (**C**) intracellular reactive oxygen species (ROS) levels of spermatozoa stored at different pH levels. Data represent mean ± SE. ^a-b^P < 0.05 versus 1 d at pH 6.2

There were no significant differences in intracellular ATP and mitochondrial membrane potential (MMP) in either pH group over 3 d (Fig. 3A and B), whereas ROS levels decreased drastically from day 2 in both pH groups (Fig. 3C).

### Acidic semen samples increased the number of female births without adverse fertility

**Fig. 4:**
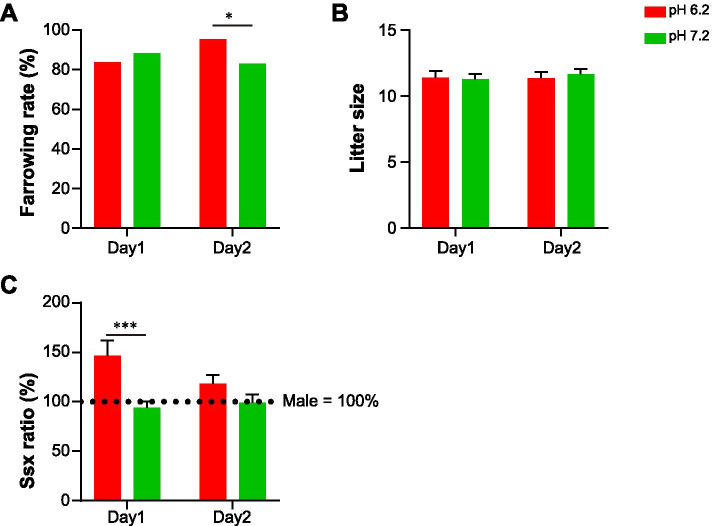
Effect of different pH levels on in vivo fertility of pig spermatozoa. Changes in the (**A**) farrowing rate, (**B**) litter size, and (**C**) relative female-to-male ratio after insemination with spermatozoa stored at different pH levels. Data represent mean ± SE. ^**^P < 0.01, ^***^P < 0.001 versus pH 7.2

To evaluate whether the use of the acidic semen samples is applicable at the farm level to increase the number of female births without fertility decline, we examined the farrowing rate, total litter size, and the female-to-male ratio. The farrowing rate was calculated as the total number of inseminated sows divided by the total number of farrowed sows in each group. In the sows inseminated on day 1, the farrowing rates were 88.31 and 83.75% for the pH 7.2 and pH 6.2 groups, respectively, and there was no significant difference between the two groups (Fig. 4A). In sows inseminated on day 2, the farrowing rate of the pH 6.2 group (95.51%) was significantly higher than that of the pH 7.2 group (83.13%) (Fig. 4A, *p < 0.01*). The acidic semen sample was not observed to have any effect on the litter size. The average litter size of the sows inseminated on day 1 was 11.29 and 11.40 in the pH 7.2 and 6.2 groups, respectively. In sows inseminated on day 2, the average litter size was 11.66 and 11.36 in the pH 7.2 and 6.2 groups, respectively (Fig. 4B). Furthermore, to determine the efficacy of acidic semen samples in producing female piglets, we analysed the relative ratio of female-to-male offspring in each group. It is noteworthy that the relative ratio of female piglets in the pH 6.2 group (146.83%) inseminated on day 1 was approximately 1.5-fold higher than that in the pH 7.2 group inseminated the same day (*p* < 0.001, Fig. 4C). Although there was no significant difference between the pH 7.2 and 6.2 groups inseminated on day 2, the relative ratio of female piglets in the litter was still higher in the pH 6.2 group (118.43%) than in the pH 7.2 group (Fig. 4C). In sows inseminated on day 1, the male ratio (5.5) was significantly higher than the female ratio (4.5) in the pH 7.2 group *(p < 0.001)*, whereas the female ratio (5.3) was significantly higher than the male ratio (4.7) in the pH 6.2 group (*p < 0.01*) (Table 1). The female-to-male ratio at pH 6.2 in sows inseminated on day 2 was 5:5; however, the male ratio was significantly higher than the female ratio at pH 7.2 on day 2 (45%: 55%, Table 1, *p < 0.001*). In addition, the number of male piglets (5.3) was significantly higher than that of female piglets (6.0) at pH 7.2 after 2 d (Table 1, *p < 0.05*). Although there was no significant difference between the pH 7.2 and 6.2 groups inseminated on day 2, the relative ratio of female piglets in the litter was still higher in the pH 6.2 group (118.43%) than that in the pH 7.2 group (Fig. 4C and Table 1).

**Table 1:** Number of male and female piglets and sex ratio after artificial insemination

		pH 7.2		pH 6.2	
		Female	Male	Female	Male
Day 1	No. of piglets	5.29 ± 0.30^a^	6.04 ± 0.22	5.88 ± 0.31	5.45 ± 0.37
	Ratio (%)	44.84 ± 1.78^***, b^	55.16 ± 1.78^b^	53.24 ± 1.80^a^	46.76 ± 1.80
Day 2	No. of piglets	5.31 ± 0.26^a^	6.32 ± 0.30	5.66 ± 0.32	5.70 ± 0.29
	Ratio (%)	44.88 ± 1.96 ^***^	55.12 ± 1.96	49.84 ± 1.72	50.16 ± 1.72

^a^Significant difference between female and male within a row at pH 7.2 and pH 6.2, respectively. ^*^ *p* < 0.05; ^***^ *p* < 0.001

^b^Significant difference in female ratios within a row between the pH 7.2 and pH 6.2 groups

Number of piglets indicates the average number of piglets per litter

Ratio (%) indicates the average of sex ratio per sows

## Discussion

Sex preselection in livestock production can improve production efficiency and provide economic benefits at the farm and industry levels. In pork meat production, although feed efficiency, growth rate, and carcass quality of the entire boar are better than those of female or castrated pigs, boars are avoided as they have a boar taint, which is the reason for an objectionable odour and taste in meat [16, 17]. Due to the increasing concerns about animal welfare, some countries have banned surgical castration without anaesthesia [18]. Therefore, producing a higher number of females than males would result in a reduction in the extra cost for castration and have a positive impact on animal welfare [19, 20]. Over the last decades, sexed spermatozoa sorted by flow cytometry according to DNA content are preferred for AI to generate the desired offspring with high accuracy [21, 22]. Generally, spermatozoa sorted by flow cytometry are stored as frozen semen samples with a small number of spermatozoa. However, this method is not applicable to porcine AI because a large number of spermatozoa per dose are required for AI and the poor quality of spermatozoa obtained after the freeze-thawing process [10]. Furthermore, the cost of equipment is one of the limitations of using this technique at the farm level [2]. Therefore, the development of a new and more efficient and simplified method for sex preselection is needed.

Our previous studies have demonstrated that X spermatozoa survive functionally longer than Y spermatozoa under stress conditions such as chemical exposure, temperature, and pH in mice and humans [11–13]. Both in previous [15] and this study, approximately 50 μL of 30% HCl was added into extended semen samples directly to adjust pH 6.1 to 6.2 for minimizing the detrimental effects during additional semen sample preparation procedure such as centrifugation and equilibration with new extender. We found that there were no differences in quantity (ATP concentration, MMP, and ROS) and quality of semen samples (farrowing rate and litter size) between control and acidic semen samples. These results indicate that direct addition of small amount of 30% HCl in the semen samples could not be quite detrimental for sperm cells. In addition, we found that the X:Y ratio of live spermatozoa in an acidic boar semen sample (pH 6.2) was maximised at 1.2:1 without a negative effect on sperm quality, such as sperm motility, viability, and capacitation status [15]. Moreover, we assessed the expression level of fertility-related biomarkers to determine the effect of storage at different pH on the male fertility [23–25]. There were no differences of protein and mRNA expressions during storage at different pH conditions. The production of female offspring may increase when acidic BTS is used for at least 2 days without negative effects on sperm fertility [15]. Based on our previous studies, we tried to confirm whether the acidic semen samples can enhance female production at the farm level. To obtain reasonable AI results, more than 300 sows were inseminated with acidic and original semen samples for 2 d. Although sperm motility, VSL, and VAP were significantly lower in acidic semen samples than in the original semen samples, no differences in the percentage of live spermatozoa were detected. Furthermore, there was no reduction in farrowing rate and litter size following AI using acidic semen samples compared to the original semen sample over 2 d. These results are in accordance with our previous report suggesting that viability can be maintained in acidic conditions without a decline in intrinsic sperm fertility [15]. Based on these results, we hypothesise that the fertilising ability of spermatozoa can be maintained in acidic semen samples similar to those in the epididymis that maintain quiescence in acidic conditions to prevent energy consumption before fertilisation [26]. Moreover, You et al. [11] found that more time-dependent increase in apoptosis and DNA damage in the Y spermatozoa under stress condition, such as alkaline pH, compared to those of X spermatozoa. Thus, we suggest that Y spermatozoa may have more vulnerable to acidic stress than X spermatozoa leads to increase the fertilization chance of X spermatozoa. Interestingly, our previous in vitro study showed that the X:Y ratio of live spermatozoa in acidic semen samples was 1.2:1 on day 1. We found that the number of female piglets was approximately 1.5- and 1.2-fold higher than that of male piglets from sows inseminated on days 1 and 2, respectively. These results may indicate that more X spermatozoa can survive and be selected during transit in the female reproductive tract than Y spermatozoa following stress exposure [27]. Moreover, these results indicated that overall fertility following the gender preselection by the decrease in the one sperm population could not effected.

## Conclusions

We hypothesise that a sufficient number of X spermatozoa for AI may present and maintain fertility in acidic semen samples, whereas stress selectively decreases Y spermatozoa. Altogether, we propose that our modified boar semen sample is simple, economical, and may enhance the productivity of female piglets at the farm level. However, as sperm fertility can only be maintained for 2 d, a more stable method is required for long-term storage without affecting the intrinsic fertility of the samples.

## Methods

### Ethical statement

All procedures were performed according to the guidelines for the ethical treatment of animals approved by the Institutional Animal Care and Use Committee of Chung-Ang University, Seoul, Korea.

### Sample preparation

Boar semen samples were purchased from Darby Genetics Inc. (Anseong, Gyeonggi-Do, Republic of Korea). Boars were housed in individual pens at 20 ± 5 °C, 2:1 light/dark cycle with continuous air circulation system. The boars were fed a commercial feeding mixture according to the nutritional requirements for male boars and free access to water. Semen samples were collected from sexually matured Duroc boars (11 to 23 months, > 90 kg body weight) using the gloved-hand method and diluted in the Beltsville thawing solution. To eliminate individual differences, three to four boar semen samples were pooled and used for evaluation of sperm functional parameters and fertility. Non-treated pooled semen samples were used as control (pH 7.2) and pH of acidic semen samples was adjusted to pH 6.2 by 30% of hydrochloric acid. The pH of control and acidic semen samples was evaluated before analysis and AI during 3 days using pH meter (Bante, China). The average pH of acidic semen samples was 6.13 ± 0.07 at day 1, 6.16 ± 0.06 at day 2, and 6.22 ± 0.08, respectively. And the average pH of control semen samples was 7.22 ± 0.01 at day 1, 7.23 ± 0.03 at day 2, and 7.23 ± 0.01, respectively. Following day 1, day 2, and day 3 incubation at 17 °C, sperm functional parameters were evaluated (*n* = 8). For artificial insemination, pooled semen samples divided into three to four vials according to number of sperm cells (30 × 10^8^/100 mL). Total twenty boars were used for the insemination.

### Sperm viability, motility, and motion kinematics

Generally, HOST is used to evaluate the functional integrity of the sperm plasma membrane, it can be used as the viability test because membrane intact spermatozoa have a positive hypo-osmolar swelling reaction that indicate viable spermatozoa [28]. To analyse the viability of spermatozoa at different pH levels, a hypo-osmotic sperm swelling test (HOST) was used as explained in our previous study [29]. In brief, the sperm samples were incubated in the HOST solution (distilled water: 0.9% w/v NaCl [1:1], 150 mOsm/kg) for 30 min at 37 °C. Next, the sperm suspension was smeared onto a glass slide, air-dried, and fixed in a fixative containing 30% methanol and 10% acetic acid in distilled water. Approximately, 500 sperm cells were analysed in random fields for each sample.

Sperm motility and motion kinematics were determined using computer-assisted sperm analysis (CASA) (SAIS-PLUS version 10.1; Medical Supply, Seoul, Korea) as previously described [30].

### Intracellular ATP

Intracellular ATP was determined using an ATP Bioluminescence Assay Kit HS II according to the manufacturer’s instructions (Roche Molecular Biochemicals, Mannheim, Germany). In brief, 25 μL of a semen sample (1 × 10^8^ sperm cells/μL) was added to a 96-well plate, followed by 25 μL of lysis buffer, and incubated for 5 min at room temperature. Next, 50 μL of luciferase reagent was added and ATP bioluminescence intensity was determined using a microplate reader (GloMax-Multi Microplate Multimode Reader; Promega, Madison, WI, USA).

### Mitochondrial activity

Mitochondrial membrane potential (MMP) was measured using JC-1 mitochondrial membrane potential assay kit according to manufacturer’s instruction (Abcam, Cambridge, MA, USA). Briefly, spermatozoa were washed with modified TCM 199 (Sigma, St. Louis, MO, USA) medium containing 10% fetal bovine serum, 0.91 mM sodium pyruvate, 3.05 mM D-glucose, 2.92 mM calcium lactate, and 2.2 g/L sodium bicarbonate (mTCM 199) at 100×g for 10 min. Next, spermatozoa were incubated with mTCM 199 containing 1 μM JC-1 for 30 min at 37 °C in the dark. And then, spermatozoa were washed by centrifugation with 1 mL of 1 x dilution buffer at 100×g for 10 min 2 times. Sperm cells were resuspended with mTCM 199 and 100 μL of a semen sample (1 × 10^6^ sperm cells/well) were added to a 96-well plate. Fluorescence was detected with a microplate fluorometer (Gemini Em; Molecular Devices, Sunnyvale, CA, USA) and analysed using SoftMax Pro 5 (Molecular Devices).

### Levels of intracellular reactive oxygen species (ROS)

To determine the intracellular ROS level, the oxidation-sensitive fluorescent dye DCFDA (Abcam, Cambridge, MA, USA) was used as described in our previous study [31]. The semen sample was centrifuged at 100×*g* for 10 min, and the sperm pellet was resuspended in 1 mL of DCFDA. After 30 min of incubation at 37 °C in an atmosphere of 5% CO_2_, the sample was washed with 1x buffer solution and resuspended in 1x supplemental buffer. Fluorescence was detected using a microplate fluorometer (Gemini Em; Molecular Devices, Sunnyvale, CA, USA) and calculated with SoftMax Pro 5 (Molecular Devices).

### Artificial insemination

The study was carried out in a Youna pig farm located in Anseong, South Korea.. To eliminate individual differences, three to four boar semen samples were pooled and then divided into three to four vials according to number of sperm cells (30 × 10^8^/100 mL). Total twenty boars were used for the insemination. Multiparous (2–8 parity) sows were used for AI to eliminate fertility variation and the age range of inseminated sows was 18 to 48 month. Heat check was performed by back-pressure test using hand within 5 days post-weaning. Two times AI were performed on day 1 and day 2 after semen storage in BTS on a total of 329 multiparous Youna sows following oestrus detection. First insemination was performed within 24 h of oestrus detection, and second insemination was completed after 24 h after first insemination. Pregnancy was detected twice by ultrasonography at 24 and 35 days after insemination. The number of sows inseminated with spermatozoa stored at pH 7.2 and those inseminated with spermatozoa stored at pH 6.2 on day 1 was 77 and 80, respectively. On day 2, 83 and 89 sows were inseminated in the pH 7.2 and pH 6.2 groups, respectively.

### Statistical analysis

Data were analysed using a two-way analysis of variance (ANOVA) with GraphPad Prism (Version 9.0; GraphPad Software Inc.). When data showed a normal distribution, comparison of the sperm functional parameters including motility, motion kinematics, sperm viability, ATP, MMP, and ROS level between group was conducted by Tukey’s multiple comparison test. Otherwise, a Mann-Whitney test was used to analyse. Chi-square test was used to evaluate the difference in the farrowing rate between the pH 6.2 and 7.2 groups inseminated on day 1 and day 2. Statistical significance was set at *P* < 0.05, and data are expressed as mean ± SEM.

## Data Availability

All data generated or analysed during this study are included in this published article.

## Abbreviations

BTS: Porcine semen extender
AI: Artificial insemination
X: X-chromosome-bearing spermatozoa
Y: Y-chromosome-bearing spermatozoa
MMP: Mitochondrial membrane potential
ANOVA: Analysis of variance
HYP: Hyperactivated spermatozoa
VSL: Straight line velocity
VAP: Average path velocity
